# Supplementary material for: Recovery of IR700 Fluorescence After Near-Infrared Photoimmunotherapy: Discovery and Mechanistic Insights
Source: Cancers (Basel). 2026 Jan 2;18(1):162. doi: 10.3390/cancers18010162 (PMC12784890; doi:10.3390/cancers18010162)
Supplement: Supplementary file 1 [file cancers-18-00162-s001.zip › cancers-4069284-supplementary.pdf]

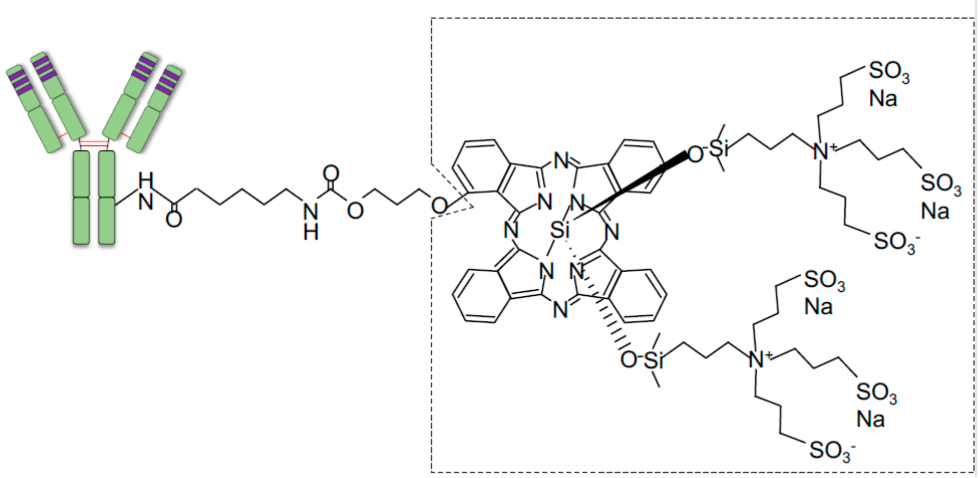

Figure S1. Chemical structure of IR700.

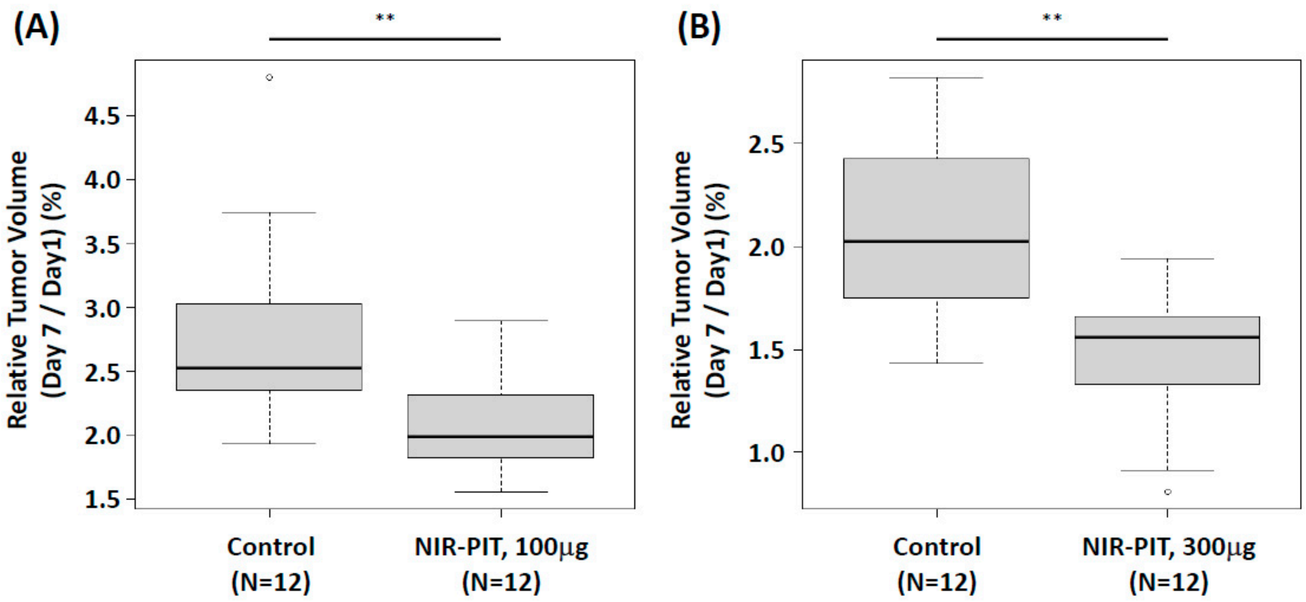

Figure S2. Relative tumor volume at day 7 compared to the one at day 1. (A) Control and NIR-PIT with cet-IR700 at 100 mg. (N =12; Mann–Whitney U test; **, $p < 0.01$; Control: 2.79±0.23, NIR-PIT: 2.07±0.11). (B) Control and NIR-PIT with cet-IR700 at 300 mg. (N =12; Mann–Whitney U test; **, $p < 0.01$; Control: 2.06±0.13, NIR-PIT: 1.47±0.10). NIR-PIT, near-infrared photoimmunotherapy.

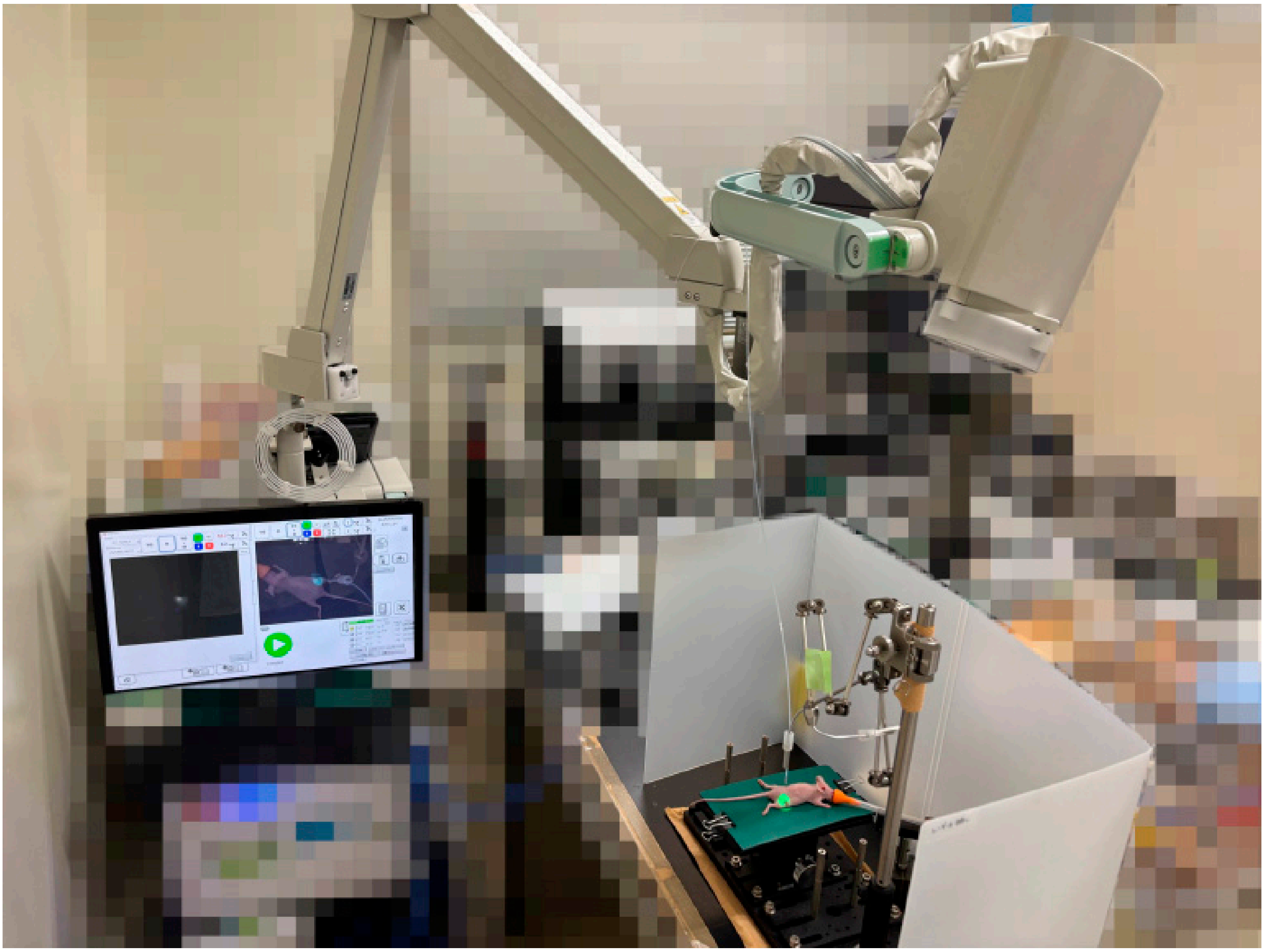

Figure S3. Experimental setup.

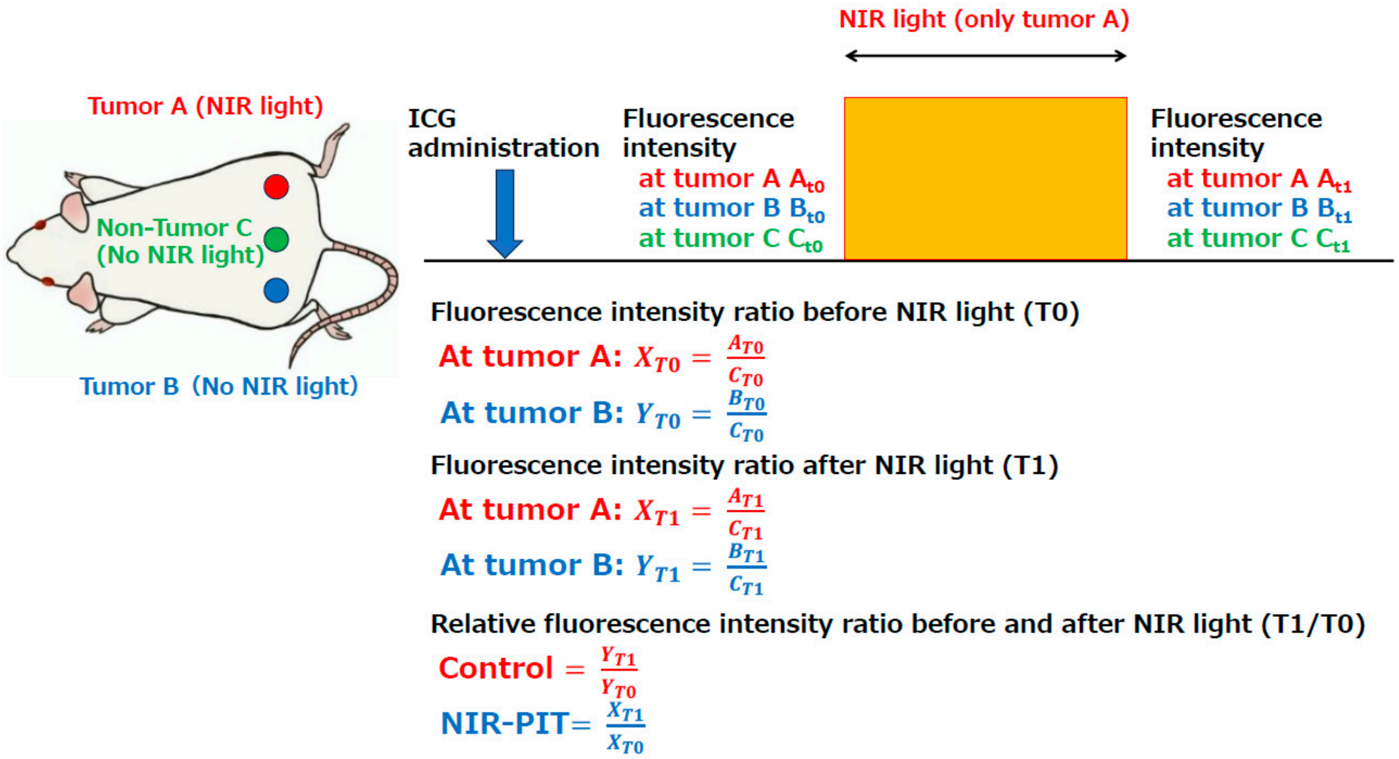

Figure S4. Method for evaluating blood flow by ICG.

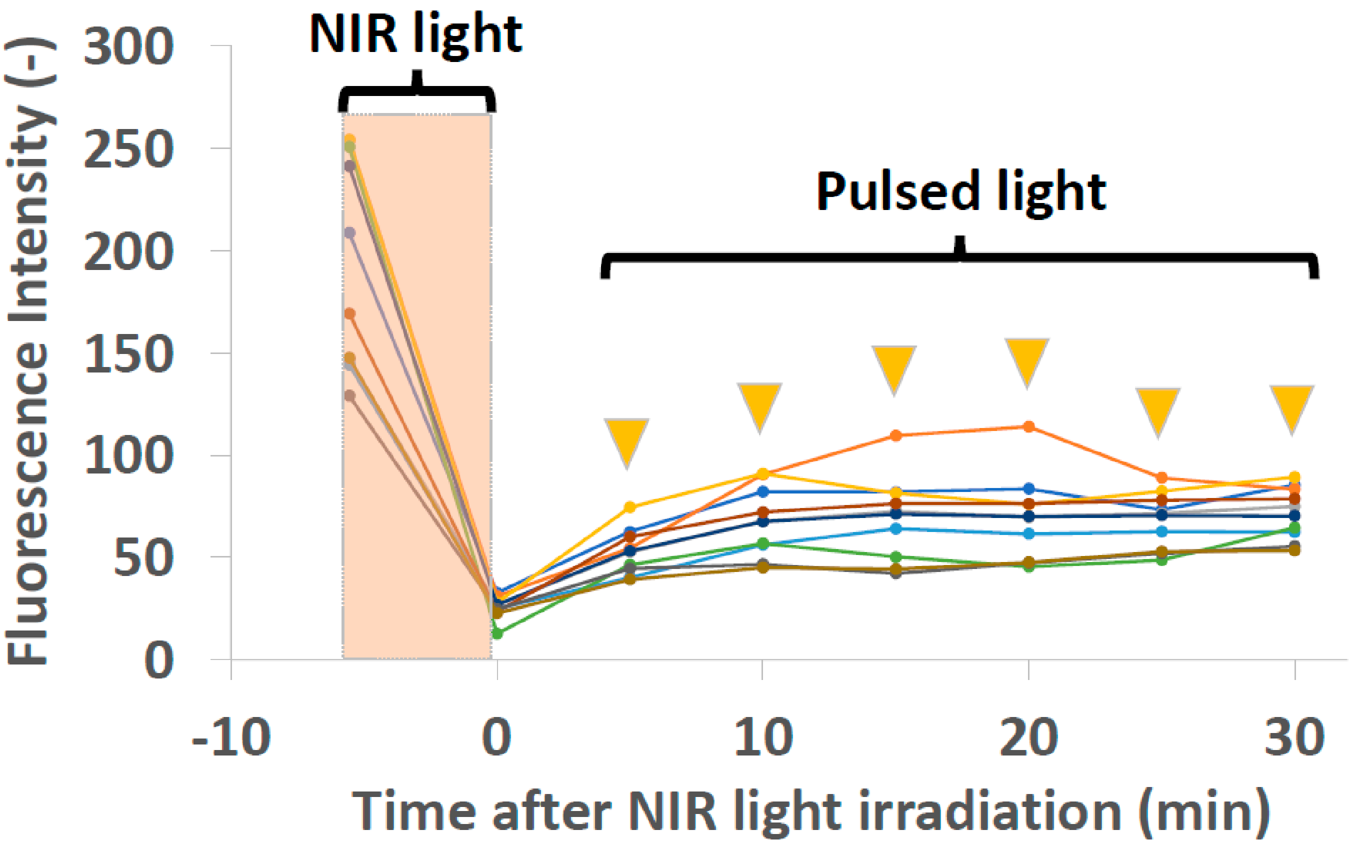

Figure S5. The change of fluorescence intensity at each time point.

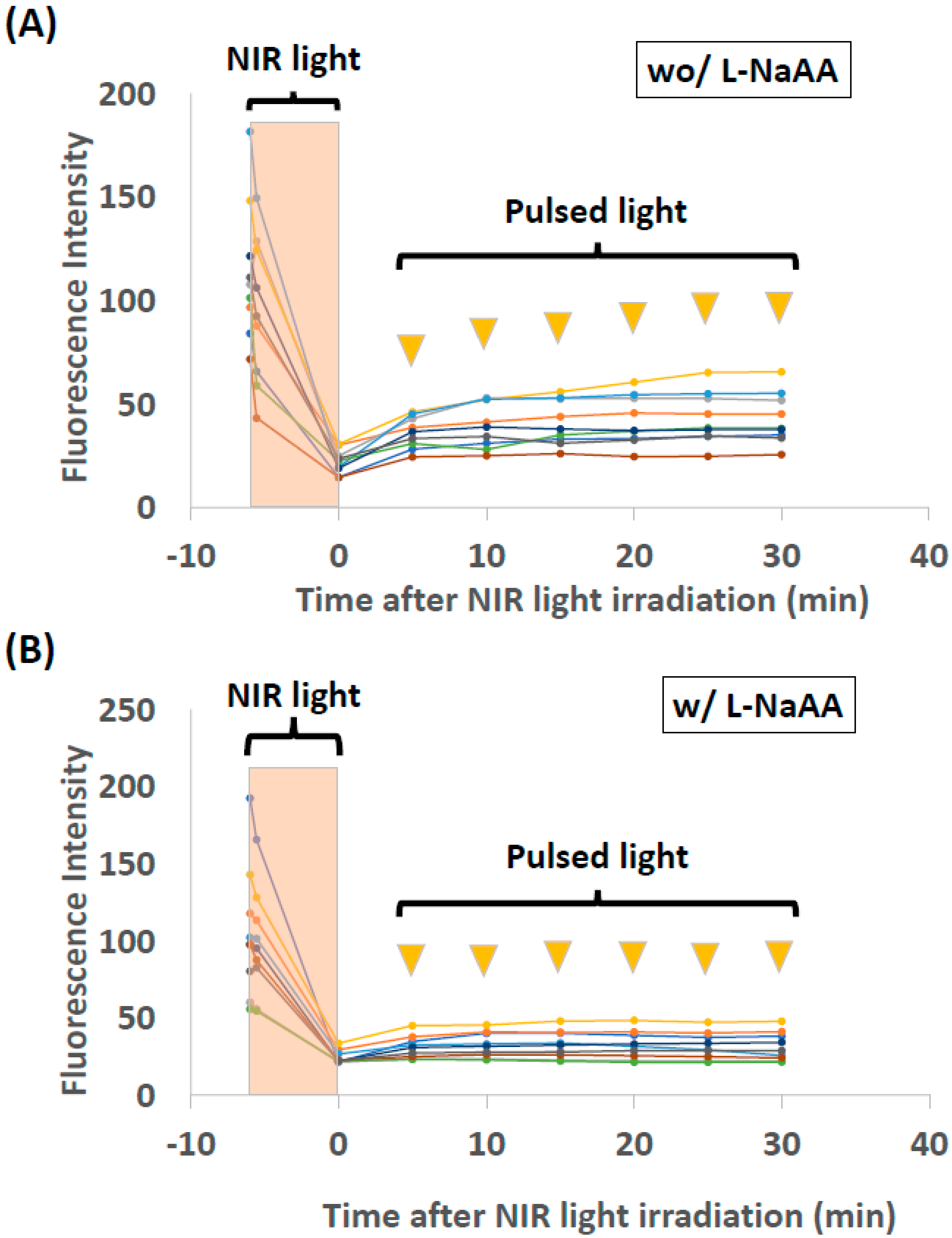

Figure S6. The change of fluorescence intensity (A) with or (B) without L-NaAA at each time point.

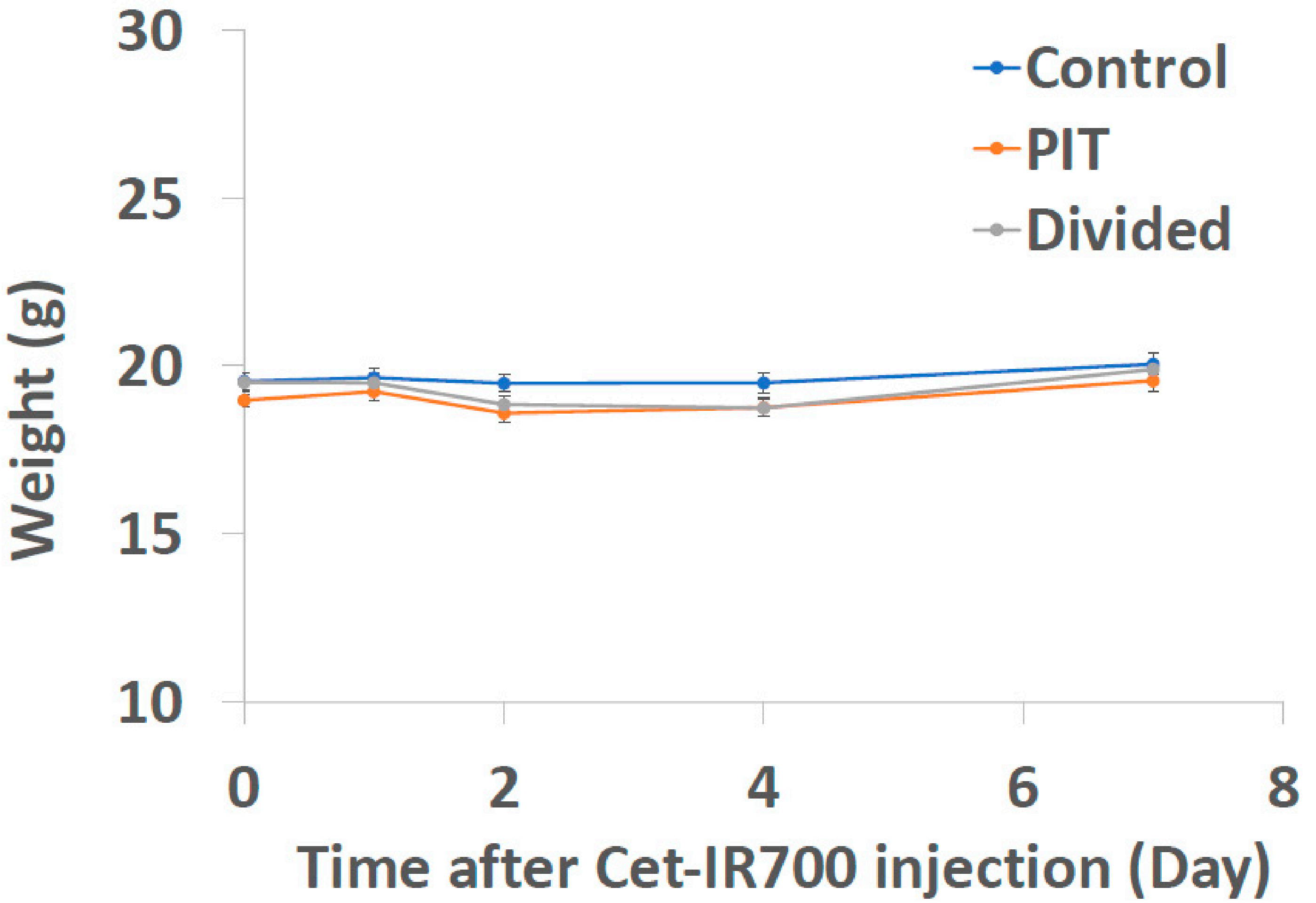

Figure S7. Changes in body weight of mice bearing A431 tumors during the experimental period.

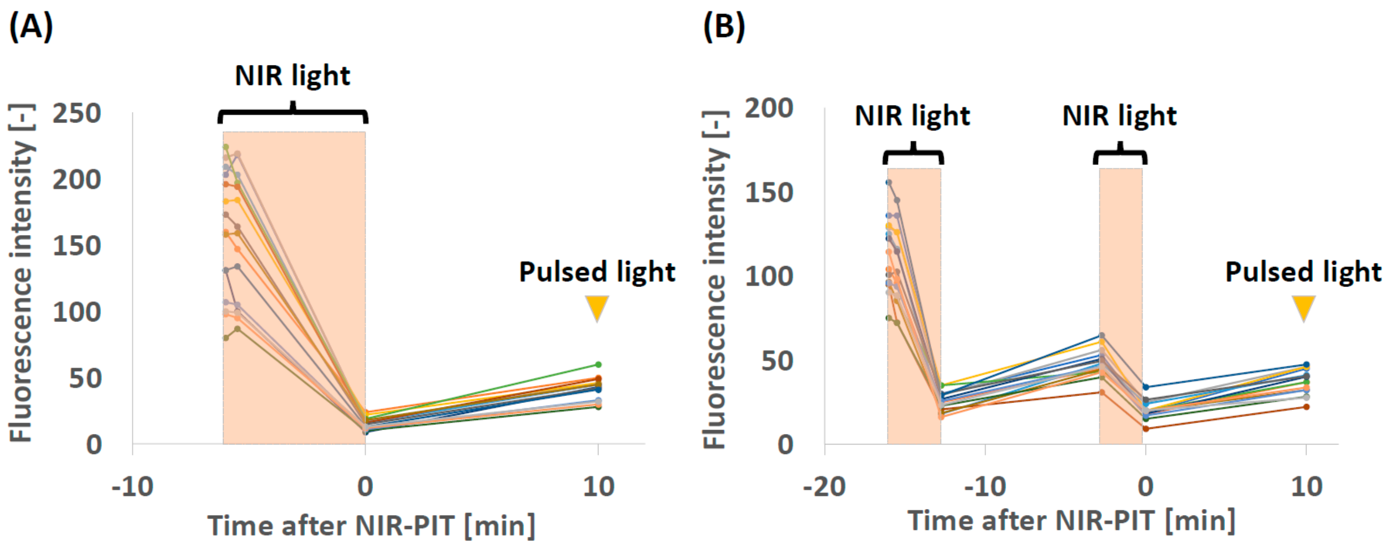

Figure S8. Fluorescence intensity curve with (A) a NIR light irradiation and (B) divided light irradiation.

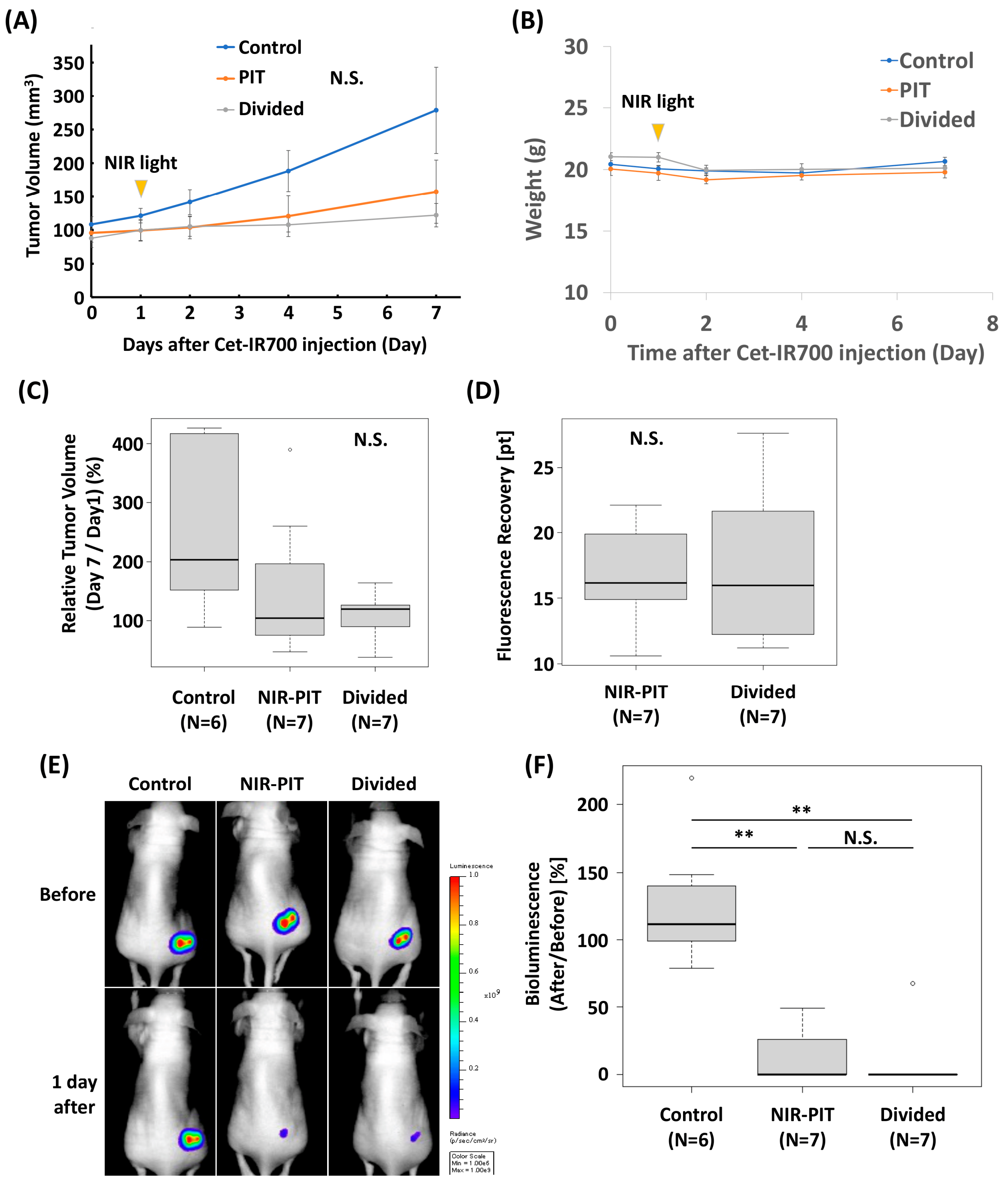

Figure S9. In vivo EGFR-targeted NIR-PIT with divided light irradiation in Fadu-Luc cells tumor-mouse model. (A) Tumor growth curve. (n = 6 at control, n = 7 at NIR-PIT and Divided; Steel-Dwass test; N.S., no significant at day 7). (B) Changes body weight. (C) Relative tumor volume at day7. (D) Fluorescence recovery of NIR-PIT and divided groups. (E) Representative bioluminescence images before and 1 day after NIR-PIT. (F) Bioluminescence signal at 1 day after against before NIR-PIT. (n = 7 at control and Divided, n = 8 at NIR-PIT; Steel-Dwass test; **, $p < 0.01$, N.S., no significant).

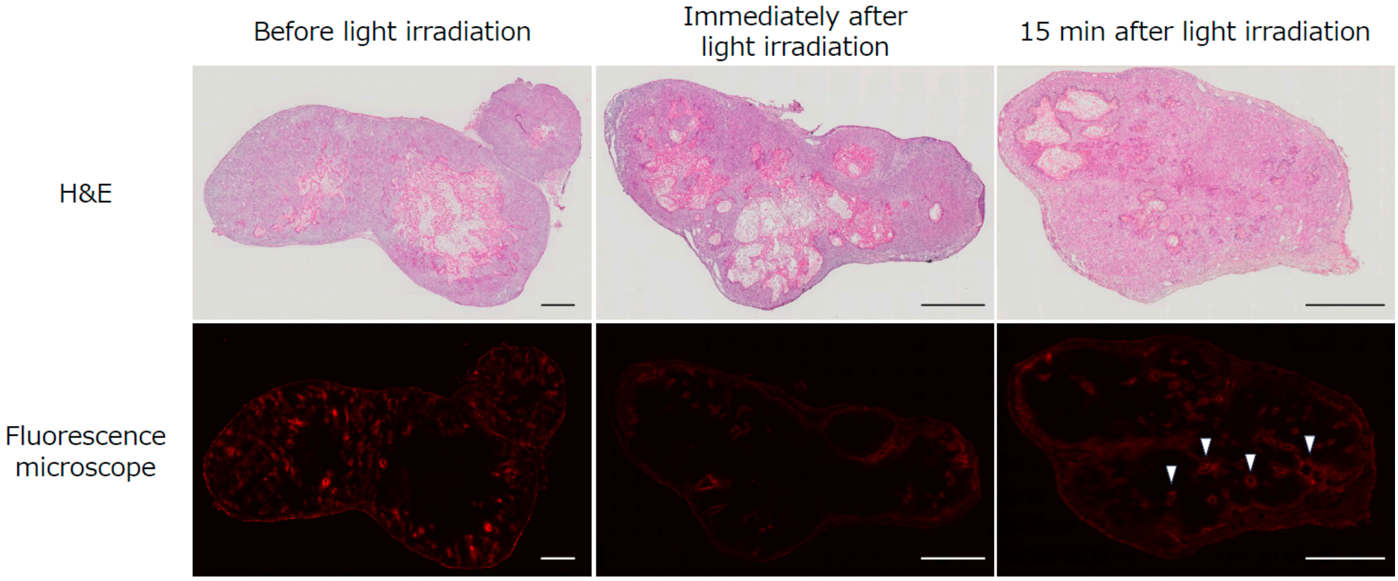

Figure S10. H&E staining and fluorescence microscopy images of tumors excised before and after light irradiation in a A431 tumor mouse model. Scale bars represent 1mm. White triangles indicate where fluorescence recovery was particularly pronounced. H&E, hematoxylin and eosin stain.
